# Diet and lifestyle interventions in postpartum women in China: study design and rationale of a multicenter randomized controlled trial

**DOI:** 10.1186/1471-2458-10-103

**Published:** 2010-02-27

**Authors:** Wei Bao, Aiguo Ma, Limei Mao, Jianqiang Lai, Mei Xiao, Guoqiang Sun, Yingying Ouyang, Shuang Wu, Wei Yang, Nanping Wang, Yanting Zhao, Juan Fu, Liegang Liu

**Affiliations:** 1 Department of Nutrition and Food Hygiene and MOE Key Lab of Environment and Health, School of Public Health, Tongji Medical College, Huazhong University of Science & Technology, 13 Hangkong Road, Wuhan 430030, PR China; 2 Institute of Human Nutrition, Medical College of Qingdao University, 38 Dengzhou Road, Qingdao 266021, PR China; 3 Department of Nutrition and Food Hygiene, School of Public Health and Tropical Medicine, Southern Medical University, Guangzhou 510515, PR China; 4 National Center for Chronic and Noncommunicable Disease Control and Prevention, Chinese Center for Disease Control and Prevention, 27 Nanwei Road, Beijing 100050, PR China; 5 Hubei Maternal and Child Health Hospital, 745 Wuluo Road, Wuhan 430070, PR China; 6 Department of chemical and biology, Medical Science College of China Three Gorges University, 8 Daxue Road, Yichang 443002, PR China

## Abstract

**Background:**

"Doing the month", or "sitting month", is a traditional practice for postpartum women in China and other Asian countries, which includes some taboos against well-accepted healthy diet and lifestyles in general population. Previous studies have shown this practice may be associated with higher prevalence of postpartum problems. The current multicenter randomized controlled trial (RCT) aims to evaluate outcomes of diet and lifestyle interventions in Chinese postpartum women.

**Methods/Design:**

The current multicenter RCT will be conducted in three representative areas in China, Shandong province, Hubei province and Guangdong province, which locate in northern, central and southern parts of China, respectively. Women who attend routine pregnancy diagnosis in hospitals or maternal healthcare centers will be invited to take part in this study. At least 800 women who meet our eligibility criteria will be recruited and randomly assigned to the intervention group (n > = 400) and the control group (n > = 400). A three-dimension comprehensive intervention strategy, which incorporates intervention measures simultaneously to individual postpartum woman, their family members and community environment, will be utilized to maximize the effectiveness of intervention. Regular visiting and follow-up will be done in both group; nutrition and health-related measurements will be assessed both before and after the intervention.

**Discussion:**

To our knowledge, this current study is the first and largest multicenter RCT which focus on the effectiveness of diet and lifestyle intervention on reducing the incidence rate of postpartum diseases and improving health status in postpartum women. We hypothesize that the intervention will reduce the incidence rates of postpartum diseases and improve nutrition and health status due to a balanced diet and reasonable lifestyle in comparison with the control condition. If so, the results of our study will provide especially important evidence for changes in both the concept and action of traditional postpartum practice in China.

**Trial Registration:**

ClinicalTrials.gov ID NCT01039051.

## Background

The postpartum period, or puerperium, which starts about an hour after the delivery of the placenta and includes the following six weeks, covers a critical transitional time for a woman, her newborn and her family, on a physiological, emotional and social level. However, the postpartum period is often neglected by maternity care in both developing and developed countries [1]. In addition, women's needs during this period have been all too often eclipsed by the attention given to pregnancy and birth [2]. The lack of postpartum care ignores the fact that the majority of maternal deaths and disabilities occur during the postpartum period and that early neonatal mortality remains high [3,4].

"Doing the month", or "sitting month", is a well-accepted and obeyed traditional practice among postpartum women and their families in China, which is also common in other Asian countries, such as Korea, Thailand, and Singapore [5-7]. According to traditional customs, women who are "doing the month" are advised to lie in bed all the time with doors and windows closed, and consume plenty of eggs or meat, drink bowels of chicken soup, brown sugar water, and millet gruel every day, while avoid eating any raw and cold food (mainly refers to fruits, vegetables), because cold food were thought to be unfavorable for postpartum recovery [8-10]. This practice seems to be believed more or less by almost all Chinese population worldwide, rather than only by local people in China mainland. Immigration studies showed that Chinese women who immigrated to Australia, Canada or United States still think that the traditional puerperium practice of "doing the month" is very important, and this belief is not subject to their level of education, acculturation level and the duration of immigration [11-14].

We have ever conducted a preliminary study in three regions of Hubei province in China to investigate prevalence of puerperium practice and their effect on postpartum women's health [15,16]. We found that 18% of women never ate vegetables, 78.8% never ate fruit and 75.7% never drank milk during the puerperium while, in contrast, the consumption of eggs and brown sugar were as high as 365.0 g/d and 81.3 g/d in rural postpartum women, which is associated with postpartum problems including constipation, backaches, breast problems, oral diseases and anemia, et al [15].

However, as indicated in a Cochrane systematic review [17], evidence is too limited at present to confirm the effect of diet and lifestyle modification on outcomes for the postpartum women and their offspring. Therefore, we initiate this multicenter randomized controlled trial (RCT) with large-scale sample size to elucidate whether a diet and lifestyle intervention can improve the health status in Chinese postpartum women.

## Methods/Design

### Objectives

This multicenter RCT is designed to assess the effectiveness of a diet and lifestyle intervention in postpartum women in China, including a short-term effect on reducing health problems during puerperium period and a long-term effect on improving health status in women and their offspring over a two-year period.

### Study Population

The current multicenter study will be conducted in three representative areas in China, Shandong province, Hubei province and Guangdong province, which locate in northern, central and southern parts of China, respectively. Women who attend routine pregnancy diagnosis in hospitals or maternal healthcare centers will be invited to take part in the randomized controlled intervention trial.

### Sample size

Sample size has been calculated as the following formula [18]:

, in which *p*_*1 *_indicates incidence rate in control group, *p*_*2 *_indicates incidence rate in intervention group and  indicates average of *p*_*1 *_and *p*_*2*_. Z_α/2 _is 1.96 when significance level (α) is 0.05 and Z_β _is 1.64 for power (1 - β) is 0.90.

Take constipation, a typical diet-related digestive disorder, for example. The incidence rate of constipation in our previous study is 20.95% in the control group [16]. We aim to reduce the incidence rate by 50%, which is 10.475%, the sample size N = = 309.89 ≈ 310 for each group. On the basis of the above calculation, a sample size of 800 participants (400 in the intervention group and 400 in the control group) has been determined, considering a certain attrition rate. This sample size is adequate, with statistical power >80%, for nearly all the major outcomes, including changes in nutritional status and health status.

### Recruitment process

**Figure 1:**
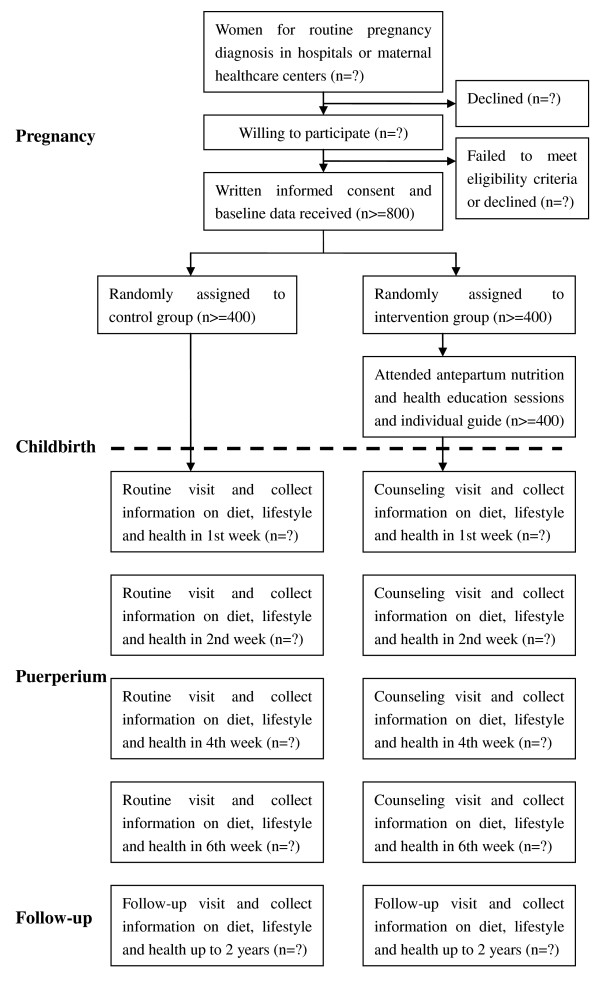
**Flowchart of participant's recruitment and trial design**.

The flowchart of participant's recruitment process and trial design is summarized in figure 1. Pregnancy women that are willing to take part in our study will be checked by our eligibility criteria. The eligibility criteria for enrollment includes: 1) healthy pregnant women; 2) at their third trimester; 3) had at least three routine examinations at these antenatal clinics [16]. A written consent form will be assigned by each woman to confirm her willingness to participate, and then baseline data will be collected by a questionnaire survey (main components shown in Table 1) and a consequent anthropometric measurement.

**Table 1:** Overview of the Questionnaires

Categories	Items
General information	Age, residence, occupation, education level, family economic situation, height, body weight, diagnosis of diseases, family history of diseases

Basic knowledge in nutrition and health	- Food guide pyramid knowledge - Nutrient-food association knowledge - Special nutritional requirement during postpartum period

Food intake	- Frequency and amount of cereals intake - Frequency and amount of vegetables intake - Frequency and amount of fruits intake - Frequency and amount of meat intake - Frequency and amount of eggs intake - Frequency and amount of milk and its products intake - Frequency and amount of soybean and its products intake - Frequency and amount of brown sugar intake - Frequency and amount of dietary oil intake - Frequency and amount of salt intake - Frequency and amount of other substance intake (alcohol, strong tea, coffee, et al.)

Health-related behaviors and Physical activities	Teeth brushing, Bathing and Hair washing Ventilation Always lie in the bed or Get up for wandering Maternal keep-fit exercises Light physical activity or Heavy physical activity Basking in the sun

**Table 2:** Comparison between Traditional Practices for "Doing the Month" and Expected Diet and Lifestyle that We Advocated during the Postpartum Period

	Traditional practices for "doing the month"	Expected diet and lifestyle during the postpartum period
**Dietary Habits**	Eat plenty of food	Increase total amount of food in accordance with energy expenditure
	
	Meat-based diet components	Diverse food with both animal foods and plant-derived foods
	
	Eat "hot" or "Yang" food (referred to meat, sugar, eggs, chicken soup, et al)	Ensure sufficient protein intake from fish, poultry meat, lean meat and eggs Enjoy milk, soybeans, or their products daily Restrict fat intake from animal foods
	
	Avoid "cold" or "Yin" food (referred to fruit, vegetables, cold water, et al)	Eat more vegetables fruits and nuts in various species Drink sufficient quantity of water and juice every day Have liquid food, such as non-greasy soup, before or between meals
	
	Keep dietary taboos in mind	Advise the woman against dietary taboos about foods which are nutritionally healthy. Reassure the mother that she can eat any normal foods. Talk to family members such as husband and mother-in-law, to encourage them to help ensure the woman eats enough.
	
		Eat more foods rich in dietary fiber
	
		Supplement with vitamins and minerals
	
		Restrict salt consumption in diet
	
		Avoid drinking alcohol, strong tea or coffee

**Other Health-related behaviors**	Never brush teeth	Brush teeth twice per day
	
	Never bathe or wash hair	If possible, take a shower every day in a warm room and wash hair at least once a week
	
	Shut well all windows or doors all the time	Ventilate regularly, but avoiding the cold wind blowing directly to the mother and child
	
	Always lie in bed without any physical activity almost all day and night	Do maternal keep-fit exercises and increase physical activities gradually to maintain a healthy weight

### Randomization

Recruited women will be randomly allocated to either intervention group or control group according to a random number generated by a computer-based procedure. Then, a unique code for each participating woman and her allocation will be recorded.

### Blinding

Although it is impossible to ensure blinding for intervention conductor and the participating women, we will ensure blinding for the outcome measurements and biochemical analyses by independent examiners unaware of group allocation. Participants cannot be blinded for the intervention, but they are asked not to reveal information about their allocation to the examiners. The key of coding concerning group assignment is only known by the programmer of the database that is used during the study.

### Interventions

On the basis of our previous study in postpartum women from Hubei province of China and the latest WHO recommendation [19], we have formed a set of recommendations for women's diet and lifestyle during the postpartum period, which is the guideline for the current intervention study. A comparison between traditional practices for "doing the month" and expected diet and lifestyle that we advocated during the postpartum period are shown in Table 1. It is obvious that our guideline emphasizes the importance of increasing consumption of vegetables and fruits, which is regard as a diet taboo for postpartum women in traditional Chinese practice. In addition, it also emphasizes the importance of maintaining energy balance through reducing excessive energy from meat, eggs and brown sugar and increasing energy expenditure from appropriate physical activity, such as doing maternal keep-fit exercises.

**Figure 2:**
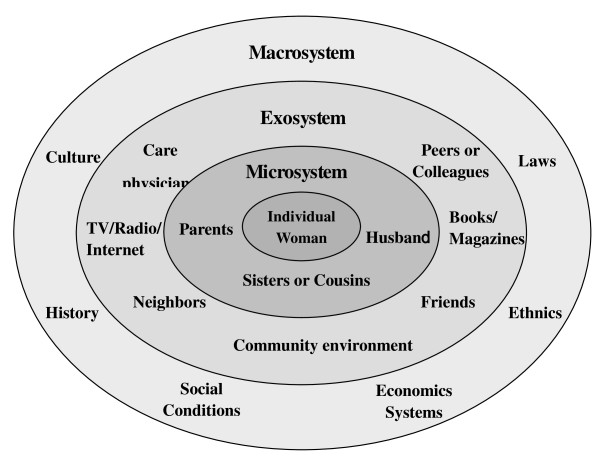
**Social Ecological Model showing potential factors that influence postpartum women's belief and practice**.

In the past decades, social ecological models have been increasingly used to formulate intervention strategies in comprehensive consideration, because simple interventions are unlikely to work on their own and the development of effective interventions requires strategies that affect multiple settings simultaneously [20]. As shown in Figure 2, a panel of environmental factors that influence the knowledge, belief and practice of postpartum women (the core) has been identified. Microsystem, mainly referred to family members of the individual woman, is crucial immediate environmental factors that should be considered in priority. Exosystem, including surrounding people, such as peers, friends, colleagues, neighbors and care physicians, and sources of knowledge, such as book, magazines, TV show, radio and internet, does not usually directly interact, but that can still affect the individual woman. In addition, we have to consider macrosystem, including history, culture, laws, ethnics, social conditions and economic systems, to give a personalized guide to individual postpartum woman. Previous studies by us and other groups have suggested that main influence factors on the diet and lifestyle of a postpartum woman include the woman's knowledge about postpartum nutrition and health care, the woman or her husband's educational level and family income levels, and the traditional concept learned from her mother in-law (49.4%), mother (34.0%), books and magazines (16.5%), relatives (14.3), friends or colleagues (12.6%) [15,21].

Therefore, we have formulated a three-dimension comprehensive intervention strategy (Table 3), which incorporates intervention measures simultaneously to individual postpartum woman, their family members and community environment to maximize the effectiveness of our multicenter trial. For individual postpartum woman, we will set antepartum participatory training courses, provide specially prepared brochures and supporting VCD, set up a specialized counseling hotline and give individual bedside guidance after birth. For their family members, we will invite every postpartum woman's husband and mother to take part in workshops or seminars, making them know some basic knowledge of postpartum care, and persuading them to promote adoption of the diet and lifestyle we advocated by the postpartum woman. For community environment, we will put up posters about basic knowledge of postpartum care in the community bulletin boards for general population educating and mobilize community leaders and heads from and maternal and child health centers for maximum support.

**Table 3:** Summary of our three-dimension comprehensive intervention strategy

Dimensions	Subjects	Strategies and Measures
1	Postpartum woman	- Participatory training for woman who expects to childbirth with 1 month - Providing specially prepared release of "Chinese women's postpartum reasonable diet and lifestyle program" (Universal Edition) and supporting VCD, to facilitate self-learning at home - Setting up a specialized counseling hotline to answer the questions from postpartum woman and their family members - Individual bedside guidance for postpartum woman, focusing on how to correctly adjust her diet and life style during postpartum period

2	Family members	Inviting every postpartum woman's husband and mother to take part in workshops or seminars, making them know some basic knowledge of postpartum care, and persuading them to promote adoption of the diet and lifestyle we advocated by the postpartum woman

3	Community support	- General population educating via putting up posters about basic knowledge of postpartum care in the community bulletin boards - Mobilizing community leaders and heads from and maternal and child health centers for maximum support

### Outcome measurements

Major measurements before and after the intervention trial are shown in Table 4. The primary outcome for the current study is the changes in the knowledge and belief about postpartum practice among the women, which expect the women in intervention group know more about the nutritional requirements in postpartum period and the potential benefit from a balanced diet with appropriate amount of animal food and abundant amount of plant-derived food. The secondary outcome for the current study is the changes in the nutritional status, including both macronutrients and micronutrients level, which reflects the real action of adopting our recommendation in their everyday food intake. The tertiary and the most important outcome is the changes in postpartum recovery and health status, including a promotion for uterine involution and a reduction of postpartum diseases such as puerperal infection, fever, constipation, hemorrhoids, anal fissure, breast disease, oral ulcers, gum bleeding, anemia, pains (headache, heel pain, back pain, leg pain, joint pain, leg cramps, etc.) and postpartum depression.

**Table 4:** Overview of the Measurements before and after the Intervention

Indexes	Assessing methods
Nutrition and health knowledge	Questionnaires

Puerperium diet and lifestyle	
Diet patterns	24-hour dietary recording and FFQ
Health-related behaviors	Questionnaires
Physical activities	Questionnaires

Assessment of nutritional status	
Macronutrients intake (protein, lipid and carbohydrate, and dietary fiber from cereals, sugar, vegetables, fruits, meat, eggs, milk, soybeans, etc.)	24-hour dietary recording and FFQ, which include both food categories and amount
Micronutrients intake	
Vitamins	HPLC for vitamin A and vitamin D, load test for B vitamins and vitamin C
Minierals	AAS for serum calcium, iron and zinc
Nutrient Metabolism	
Blood glucose and lipid profiles, including total cholesterol, HDL-C, LDL-C, triglycerides	Automatic biochemical analyzer

Postpartum recovery and health status	
Anthropometric measurements, including height, body weight, and BMI	BMI = body weight (kg)/height (m^2^)
Body recovery, including duration for lochia, amount and duration of postpartum hemorrhage, degree of uterine involution	Diagnosis by specialized obstetricians
Diseases prevalence, including puerperal infection, fever, constipation, hemorrhoids, anal fissure, breast disease, oral ulcers, gum bleeding, anemia, pains (headache, heel pain, back pain, leg pain, joint pain, leg cramps, etc.)	Diagnosis by specialized doctors
Postpartum depression	Assessed by the Edinburgh Postnatal Depression Scale

Milk secretion and neo development	Assessed by specialized doctors

FFQ indicates food frequency questionnaires; HPLC, high performance liquid chromatography; AAS, atomic absorption spectrophotometry; HDL-C, high density lipoprotein cholesterol; LDL-C, low-density lipoprotein cholesterol; BMI, body mass index

### Data management and quality assurance

Data entry will be independently done by two researchers, and then a computer software-based error detection will be carried out to check the consistency of these data. If inconsistency occurs, the original questionnaire will be referred and the error will be corrected via a re-entry.

### Ethical approval

This study will be carried out in accordance with requirements documented in the Declaration of Helsinki. Ethics approval has been obtained from the local Health Department and the research ethics boards of Tongji Medical College, China.

### Statistical methods

Descriptive statistics will be calculated and checked for balance in intervention and control groups in demographic, health and outcome measurements at baseline. Comparisons between intervention group and control group will be performed by Chi-square (categorical variables), t test (continuous variables, normal distribution) or Mann-Whitney U test (continuous variables, skewed distribution). Final analyses will be undertaken using generalized linear mixed models to investigate changes over time in the two groups in the major outcomes. These models allow for repeated measures and can be used for normally distributed, binomial, and ordinal data [22]. All statistical analyses will be performed using SPSS 12.0 statistical software package (SPSS Inc, Chicago, IL, USA).

## Discussion

It is generally accepted that a balanced diet comprising abundant intake of whole cereals, fruits, vegetables and a suitable amount of fish, meat, eggs and milk is essential for maintaining our health. However, postpartum women in China are traditionally advised to follow a special dietary and lifestyle pattern, which is quite different from the dietary and lifestyle patterns they maintain before and after postpartum period [15]. Several studies have paid attention to explore the relationship of postpartum practice and consequent health problems in Chinese women [23,24]; however, as we know, it is hard to draw a clear causation in cross-sectional studies.

To our knowledge, this study is the first and largest multicenter RCT which focus on the effectiveness of diet and lifestyle intervention on reducing the incidence rate of postpartum diseases and improving health status in postpartum women. This evidence-based study is designed on the basis of data from our previous study in three regions in Hubei province of China [15,16], incorporating with the latest WHO recommendations [19]. The major limitation of this study is that the participants that we will recruit are mainly Chinese Han women, whereas the postpartum practices vary among different national minorities in China. Further research on different minority populations will be very interesting.

We hypothesize that the intervention will reduce the incidence rates of postpartum diseases and improve nutrition and health status due to a balanced diet and reasonable lifestyle in comparison with the control condition. If so, the results of our study will provide especially important evidence for changes in postpartum diet and lifestyle. The fantastic reform in both the concept and action of traditional postpartum practice in China will be expected.

## List of abbreviations

RCT: randomized controlled trial; WHO: world health organization

## Competing interests

The authors declare that they have no competing interests.

## Authors' contributions

WB and LL is responsible for developing the intervention protocol and drafting the manuscript. LL originated the idea for the study, led on its design, and supervised the project. LL, AM and LM directed the multi-center trial in Central, Northern and Southern China, respectively. JL contributed in statistical analysis and quality control of the trial. MX, GS, YO, SW, WY, NW, YZ, JF contributed in participants recruitment and data collection. All authors participated in discussing the design of the study and developing the research protocols. All authors read and corrected draft versions of the manuscript and approved of the final manuscript.

## Pre-publication history

The pre-publication history for this paper can be accessed here:

http://www.biomedcentral.com/1471-2458/10/103/prepub
